# Molecular dynamics simulations of the Nip7 proteins from the marine deep- and shallow-water Pyrococcus species

**DOI:** 10.1186/s12900-014-0023-z

**Published:** 2014-10-15

**Authors:** Kirill E Medvedev, Nikolay A Alemasov, Yuri N Vorobjev, Elena V Boldyreva, Nikolay A Kolchanov, Dmitry A Afonnikov

**Affiliations:** 1 Institute of Cytology and Genetics SB RAS, Prospekt Lavrentyeva 10, Novosibirsk 630090, Russia; 2 Institute of Chemical Biology and Fundamental Medicine SB RAS, Prospekt Lavrentyeva 8, Novosibirsk 630090, Russia; 3 Novosibirsk State University, Pirogova str. 2, Novosibirsk 630090, Russia; 4 Institute of Solid Chemistry and Mechanochemistry, SB RAS, Novosibirsk 630090, Russia; 5 NRC Kurchatov Institute, 1, Akademika Kurchatova pl., Moscow 123182, Russia

**Keywords:** Molecular dynamics simulation, Nip7 protein, High pressure, Adaptation, Salt bridges

## Abstract

**Background:**

The identification of the mechanisms of adaptation of protein structures to extreme environmental conditions is a challenging task of structural biology. We performed molecular dynamics (MD) simulations of the Nip7 protein involved in RNA processing from the shallow-water (*P. furiosus*) and the deep-water (*P. abyssi*) marine hyperthermophylic archaea at different temperatures (300 and 373 K) and pressures (0.1, 50 and 100 MPa). The aim was to disclose similarities and differences between the deep- and shallow-sea protein models at different temperatures and pressures.

**Results:**

The current results demonstrate that the 3D models of the two proteins at all the examined values of pressures and temperatures are compact, stable and similar to the known crystal structure of the *P. abyssi* Nip7. The structural deviations and fluctuations in the polypeptide chain during the MD simulations were the most pronounced in the loop regions, their magnitude being larger for the C-terminal domain in both proteins. A number of highly mobile segments the protein globule presumably involved in protein-protein interactions were identified. Regions of the polypeptide chain with significant difference in conformational dynamics between the deep- and shallow-water proteins were identified.

**Conclusions:**

The results of our analysis demonstrated that in the examined ranges of temperatures and pressures, increase in temperature has a stronger effect on change in the dynamic properties of the protein globule than the increase in pressure. The conformational changes of both the deep- and shallow-sea protein models under increasing temperature and pressure are non-uniform. Our current results indicate that amino acid substitutions between shallow- and deep-water proteins only slightly affect overall stability of two proteins. Rather, they may affect the interactions of the Nip7 protein with its protein or RNA partners.

## 1 Background

High temperatures and pressures cause damage to living cells. For humans and the best studied organisms, conditions with temperature close to 27°C (300 K) and atmospheric pressure around 0.1 MPa are optimal. However, there exist organisms, which colonize habitats extreme, life-incompatible for humans. Such conditions are near deep hot springs colonized by communities of organisms, the extremophiles [1]. Their life is sustained under conditions with temperature as high as 100°C (373 K) and pressure above 20 MPa, exceeding the atmospheric by 200 times. The mechanisms by which cell survival is provided are not well understood. Their elucidation would provide answers to some fundamental questions on the origins of life and the early adaptation of microorganisms [2], also on adaptation to the conditions of diverse ecological niches [3]. A timely challenge is the identification of the molecular mechanisms of the evolutionary adaptation of the genomes and proteomes of the living beings to the conditions of high temperature [4]-[8] and pressure [9]-[12]. Extremophiles were supposed to provide unprecedented opportunities for biotechnological explorations (single enzyme catalysis) [13]-[16]. Indeed, based on research on extremophiles, enzymes were developed for biotechnological applications.

To study the possible mechanisms of the influence of high temperature and pressure on the protein dynamics and protein adaptation to altered environmental pressure, we used here computer MD simulations of two homologous Nip7 proteins from hyperthermophilic (optimal growth temperature close to 100°C) archaea, shallow water *P. furiosus* (hydrostatic pressure close to atmospheric) [17] and deep-water, *P. abyssi* (hydrostatic pressure close to ~20 MPa) [18],[19]. The goal of our work was to compare the dynamics properties of the models we built at high and low temperatures, also at atmospheric and high pressures to identify their common characteristics, also their differences presumably related to the different depths of the organisms’ habitats.

Nip7 was initially identified in yeast as required for processing of the 27S pre-rRNA to form the mature 25S and 5.8S rRNAs [20]. It localizes to the nucleolus but was also found to sediment in the region of free 60S subunits in sucrose density gradients [20], which is consistent with its presence in pre-60S complexes [21]. Experimental evidence suggests that the *P. abyssi* Nip7 may be an exosome regulatory factor. It binds preferentially to U- and AU-rich RNAs and strongly inhibits the exosome due to its association with both the exosome complex and the substrate RNA [22].

**Figure 1:**
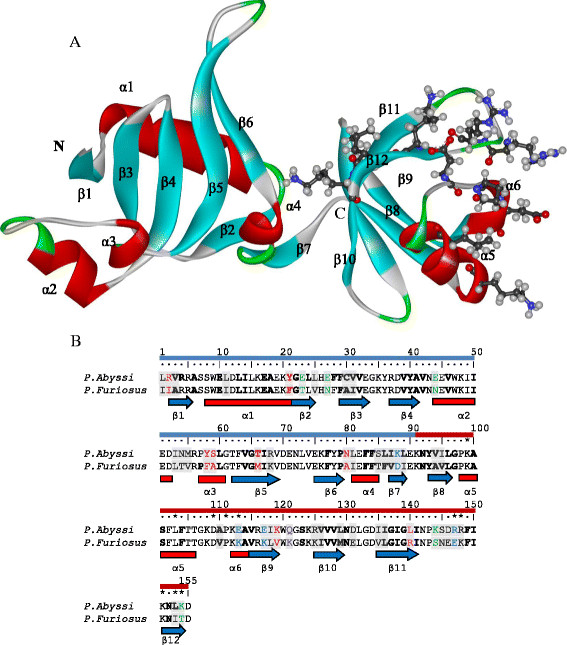
**Nip7 protein structure. (A)** 3D representation of the *P. abyssi* protein structure (2P38:A) [23]. The secondary structure elements are lettered and colored (helices red, *β*-strands blue, turns green). The N-terminal domain left, C-terminal domain right. Amino acid residues assumed to bind an RNA molecule [23] shown as ball and stick representation. **(B)** Alignment of the *P. abyssi* and *P. furiosus* sequences. The substitutions in the *P. furiosus* relative to the *P. abyssi* protein are on gray background. Distinguished are the following types of substitutions resulting in replacement of: a polar residue in *P. abyssi* by a nonpolar in *P. furiosus* (red); a charged *P. abyssi* amino acid by an uncharged in *P. furiosus* at retained polarity (green); polar amino acid in *P. furiosus* by nonpolar in *P. abyssi* (pink); *P. furiosus* charged side group by an uncharged (lilac); those resulting in oppositely charged residues (blue). Secondary structure is shown below sequences according to 2P38:A: the helices are indicated in red rectangles, blue arrows indicate *β*-strands. Residues belonging to the interior of the protein according to the GetArea web-server [27] are shown in bold letters. The symbol *denotes amino acids involved in RNA binding [23]. The N-terminal domain beneath the row of position numbers, blue; the C-terminal domain, red.

The 3D structure of *P. abyssi* Nip7 protein is known (PDB ID 2P38; Figure 1A). The protein polypeptide chain is 166 amino acids long of which 155 residues are represented in the 3D structure. The protein consists of two *α-β* domains [23], Figure 1. The N-terminal domain (residues 1–90) is composed of five antiparallel *β*-strands surrounded by three *α*-helices and one 3_10_ helix. There is an assumption that archaeal Nip7 may interact with exosome via its N-terminal domain, thereby controlling the exosome function [22]. However, the molecular mechanism of this interaction is unknown.

The C-terminal domain is assigned to the PUA class. [24]. It includes amino acid residues 91–155 and is comprised of a mixture of *β*-sheets, one *α*-helix, and one short 3_10_ helix [23]. This domain, named after pseudouridine synthases and archaeosine-specific transglycosylases [25], was initially described in tRNA modifying enzymes and in pseudo-uridine synthases from Archaea and eukaryotes [26], and it has been proposed to mediate protein-RNA interactions. Comparative structural analyses revealed that the residues involved in RNA contacts are conservative in the archaeal PUA domains [23]. In the *P. abyssi* Nip7, these are residues K103, L107, D113, P115, E117, R151, R152, K155, L157, K158 (Figure 1), whose interactions with RNA have been confirmed by the results of mutation experiments [23]. The PUA domain contacts the RNA molecule using a glycine-containing loop, which connects the fifth *α*-helix and the *β*_9_ strand via residues of the *β*_12_ strand (Figure 1). The structural alignment suggests that Nip7 may use a mechanism similar to ArcTGT for RNA interaction, which binds to the bottom of the tRNA^Val^ acceptor stem through the major groove. In the yeast and human Nip7, however, some RNA-binding residues are replaced by a glycine and three residues with hydrophobic side chain [23].

The Nip7 from *P. abyssi* and its *P. furiosus* homolog share 70% identity in their sequences (Figure 1B). Comparative analysis identified the excess of radical versus conservative amino acid substitutions fixation rates in Nip7 *P. furiosus* after divergence from the deep-sea ancestor it shared with *P. abyssi* and *P. horikoshii*[27]. It was suggested that Nip7 and some other proteins, which were concerned predominantly with “translation machinery” and “ribosomal function” evolved under positive Darwinian selection, resulted from their adaptation to altered conditions of elusive pressures. However, the molecular mechanisms underlying this mode of gene evolution remained unclear.

A deletion/insertion free alignment is an advantage for simulations of the *P. furiosus* 3D structure allowing to omit the reconstruction of loops. The Nip7 proteins are simulated at atmospheric pressures, also at 50 and 100 MPa. Given the fact that the temperatures optimal for these two organisms are close to 373 K, we consider simulations at room (300 K) and elevated (373 K) temperatures.

The results show that the models of the two proteins at all the examined pressures and temperatures are compact, stable and similar to the crystal Nip7 structure. Analysis of protein structure dynamics at different pressures and temperatures allowed us to disclose similarities and differences between the deep- and shallow-sea protein models at different temperatures and pressures.

## 2 Results

### 2.1 Stability of models

**Figure 2:**
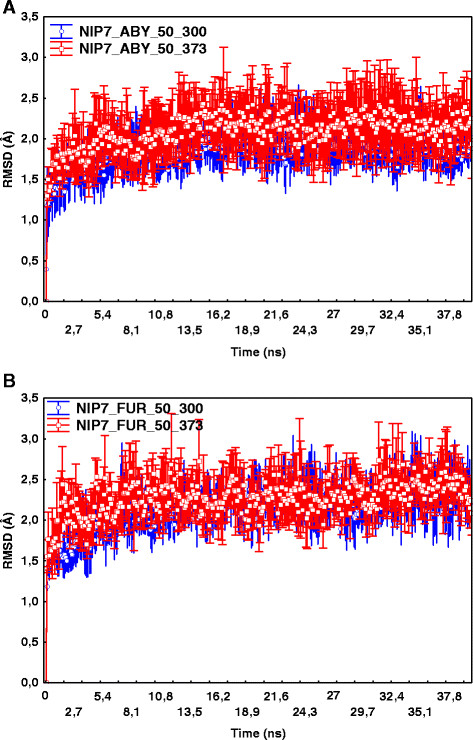
**Dependencies of the C**_**α**_**atoms RMSDs relative to the starting structure on the MD simulation step.** Points indicate RMSD values (Y axis) at the simulation time (X axis) averaged over 5 trajectory runs. Whiskers indicate the 95% confidence intervals. **(A)** RMSD for NIP7-ABY model at 50 MPa and 300 and 373 K. **(B)** RMSD for NIP7-FUR model at 50 MPa and 300 and 373 K.

Figure 2 shows changes in the root mean – square deviations (the RMSDs) of the protein C_α_ atoms relative to the starting structures during the MD simulations for the trajectories at 50 MPa. From Figure 2 it follows that the structures achieve equilibrium starting from about 10 ns of the trajectory periods. The deviations from the starting structure are about 2 Å for the NIP7-ABY model, they are about 2.25 Å for the NIP7-FUR model.

We compare the structures during simulations of the two protein models with the crystal structure of the *P. abyssi* Nip7 (2P38:A). Table 1 gives the mean RMSD values and the 95% confidence intervals at different temperatures and pressures calculated using five MD trajectories for each parameter set.

**Table 1:** **Comparison of the C**_**α**_**atom RMSDs in the NIP7-ABY and NIP7-FUR models from their positions in the crystal structure of the** ***P. abyssi*** **Nip7 (2P38:A)**

**Model**	**NIP7-ABY**		**NIP7-FUR**	
**Pressure (MPa)**	**Temperature 300 K**	**Temperature 373 K**	**Temperature 300 K**	**Temperature 373 K**
0.1	1.76 ± 0.04	1.93 ± 0.04	2.08 ± 0.03	2.41 ± 0.02
50	1.81 ± 0.04	1.94 ± 0.06	2.2 ± 0.05	2.2 ± 0.03
100	1.79 ± 0.06	1.78 ± 0.03	2.18 ± 0.02	2.23 ± 0.02

The average values and the 95% confidence intervals at different pressure and temperature values are given in Å.

As shown in Table 1, the protein deviates from the crystal structure at higher temperatures; however, the mean RMSD is not greater than 2.41 Å for NIP7-FUR and 2.0 Å for NIP7-ABY, thereby supporting the inference that the structures are stable throughout the MD simulations at all the parameters under study. Table 1 also shows that the RMSD for both proteins tends to increase with rising temperature.

**Figure 3:**
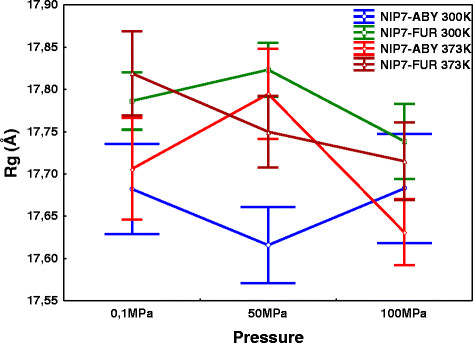
**Dependencies of the radius of gyration (Rg, Y axis) on pressure (X axis) at high and low temperatures for the Nip7 protein models NIP7-ABY and NIP7-FUR.** Whiskers indicate the 95% confidence intervals. Blue, the NIP7-ABY model, 300 K; green, the NIP7-FUR model, 300 K; red, the NIP7-ABY model, 373 K; brown, the NIP7-FUR model, 373 K.

The radius of gyration (Rg) is another indicator of stability of a structure during simulation. The average Rgs of the protein structures for trajectories at different pressures and temperatures are compared in Figure 3. As shown in Figure 3, most differences between the Rg values for different trajectories do not exceed the standard errors of means, thereby providing evidence that our models are stable. It should be noted that this is associated with somewhat greater Rg for NIP7-FUR than NIP7-ABY. Two-way ANOVA of the Rg values for NIP7-FUR and NIP7-ABY demonstrated that alterations in pressure and temperature did not result in significant changes in the mean Rg values for both protein models (Additional file 1).

### 2.2 Analysis of solvent accessibility

**Figure 4:**
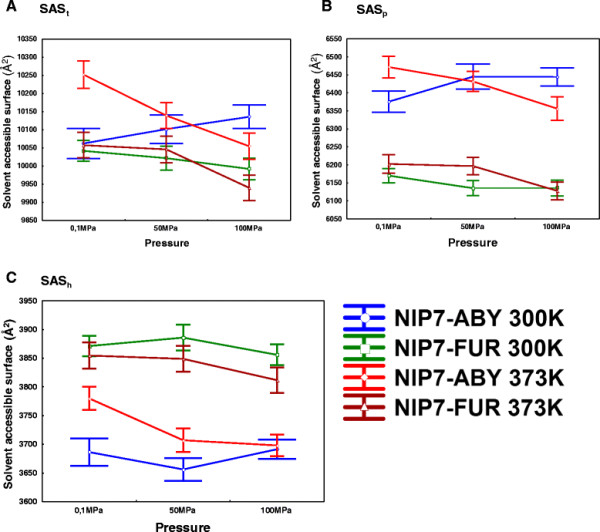
**Graphs showing the dependencies on different conditions of MD simulations for the solvent-accessible residue areas. (A)** total; **(B)** polar; **(C)** hydrophobic. Pressure is plotted along the X, the SAS area in Å^2^ along the Y axis. Whiskers indicate the 95% confidence intervals. Blue, the NIP7-ABY model, 300 K; green, the NIP7-FUR model, 300 K; red, the NIP7-ABY model, 373 K; brown, the NIP7-FUR model, 373 K.

We compute for each trajectory the average values of solvent accessibility of the residues for the entire protein (SAS_t_), also separately for the polar (SAS_p_) and hydrophobic (SAS_h_) surface fractions. We calculate the mean and standard error of these parameters over five runs for each protein model and pressure-temperature values set. The results are given in Figure 4(A-C).

As seen in Figure 4, the total area is somewhat smaller for NIP7-FUR. Higher values for the solvent accessible polar portion of the residues are observed for the NIP7-ABY than for the NIP7-FUR model (compare the blue/red line values for NIP7-ABY with the brown/green values for NIP7-FUR in Figure 4B). The area of the hydrophobic part of the model is smaller (the same lines in Figure 4C). These observations concerned all the pressure and temperature parameters we studied.

The following trends in the values of the SAS parameters of the NIP7-FUR model are notable: (1) the rise in temperature at the same pressures increases the solvent accessibility of the polar portion of the protein (SAS_p_ values for NIP7-FUR at 373 K, brown, are higher than the for NIP7-FUR 300 K, green, with the exception of 100 MPa; Figure 4B) and decreases the area of the hydrophobic portion of the surface, this is associated with a small change in the total area (Figure 4С); (2) the increase in pressure systematically reduces the SAS values (brown and green lines, Figure 4B, С). However, most changes are in the range of the mean standard errors.

As for the NIP7-ABY model, the behavior of its SAS parameters was ambiguous. SAS_p_ at atmospheric pressure and 373 K is greater, than at 300 K, whereas at 100 MPa, on the contrary, is lower (compare the red/blue line Y values, Figure 4B). A significant increase in SAS_h_ at elevating temperature (red line is above blue one, Figure 4C; 100 MPa was an exception) was other characteristic of this model. At the constant high temperature, increase in pressure resulted in decrease in the SAS values of all the three types (red lines on Figure 4A, B, C). In contrast, at constant T = 300 K, the SAS_p_ values increased with increasing pressure, although within the limits of standard deviations (blue line, Figure 4B). Changes of SAS_h_ for the NIP7-ABY model demonstrates no systematic trend at increasing pressure (blue line, Figure 4C); as for SAS_h_, it slightly changes at P = 50 MPa, as compared with P = 0.1 MPa, it rises slightly with pressure increasing to 100 MPa, becoming actually equal to SAS_h_ at P = 0.1 MPa.

We estimated the influence of temperature and pressure on change in the surface area of the models on the basis of two-way ANOVA (Additional file 2). Taken together, the data agree with the analysis shown in Figure 4. Temperature exerts a significant influence on the hydrophobic part of the NIP7-ABY surface area, thereby demonstrating that the influence of pressure and temperature on change in the total area of protein surface is not additive. This presumably reflects the difference in the SAS values between the high and low temperature NIP7-ABY trajectories. As for the NIP7-FUR model, pressure exerts a significant influence on both the hydrophobic part of the area and SAS_t_.

Thus, pressure and temperature differently affect residue solvent accessibility for the two protein models. Pressure results in a decrease in the solvent accessible surface area. This effect is the most conspicuous at high temperatures.

### 2.3 Local structure of the polypeptide chain

The next step was to define how the conformation of the different regions of the polypeptide chain deviates from the X-ray Nip7 structural model. For different trajectories, we built graphs showing the dependencies of the local structure deviation (RMSDL) parameter (see Methods). These graphs concerned every residue; we estimated also the mean RMSDL error, which characterizes its fluctuation during the simulations. (Additional file 3: Figure S1). The graphs give prominence to the non-uniform local changes in the protein structure during the simulations. There are regions where deviations are quite small (~1 Å), while in the other regions changes in the local chain conformation relative to the crystal structure are considerable (~4 Å). In such cases, the RMSDLs of the polypeptide chain conformation are greater in those regions, which correspond to the loops connecting the secondary structure elements. Regions with great RMSDL values are, as a rule, located where amino acids substitutions are numerous (see Additional file 3: Figure S1A). As a result, the evolutionary and structurally variable regions are virtually the same (with the exception of positions 85–95 making up the segment between the *α*_4_-*α*_5_ helices, Additional file 3: Figure S1A). For this segment, the RMSDL values are low, whereas the number of differences between the *P. abyssi* and *P. furiosus* Nip7 sequences is high.

Other features concern the differences in the RMSDLs values between the N- and C-terminal domains [23]. There are regions with small RMSDL values and peaks in the loop regions (positions 30–40, which correspond to the *β*_3_-*β*_4_ loop, positions 49–59 and 69–79) for the N-terminal domain. The graphs of structural variations in the C-terminal, the PUA-domain, also contain maxima at positions 105–110, 115–125, 143–148 (Additional file 3: Figure S1) and minima; however, the minima values are substantially higher than those for the N-terminal domain. From comparisons of the deviation graphs for the different trajectories, it is evident that the regions corresponding to the RMSDL maxima are by and large the same for the trajectories that correspond to both high pressures and temperatures (Additional file 3: Figure S2). This means that the local changes in the conformation of the protein chain in our models occur in the same region of the structure despite the different nature of the destabilizing factor (changes in pressure or temperature).

The effects of pressure and temperature on changes in the conformation of the polypeptide chain in the Nip7 models are now considered. The most conspicuous fact is that, at the same pressure, elevated temperature causes an increase in the RMSDLs for some protein residues (compare Additional file 3: Figure S1, panel pairs A-B, C-D, E-F). The changes in the RMSDLs of the polypeptide chain resulting from elevated temperature occur non-uniformly. For example, greater RMSDLs at elevated temperature are observed in the NIP7-ABY model in the region of the *α*_2_-*α*_3_ loop (at positions 49–59). Conversely, for some regions in this model, an increase in temperature results in their decrease, for example residues 109–127 of the C-terminal domain. A similar pattern is observed for this model at low (Additional file 3: Figure S1, panels A-B), moderate (C-D) and high (E-F) pressures. However, for some regions of the protein changes in the RMSDLs at elevated temperature are only slight.

The interesting features brought out by comparing the two proteins are the higher RMSDL values for the C-terminal domain in NIP7-FUR compared to NIP7-ABY at any pressure-temperature parameter (Additional file 3: Figure S1).

To make the local structure changes more prominent with respect to the temperature and pressure, we performed an analysis of the sensitivity of the residue RMSDL parameter to temperature (*S*^*t*^_*i*_) and pressure (*S*^*p*^_*i*_) expressed as the ratio of the RMSDL at high and low temperature and high and low pressure (see Methods). We also estimated the dependence of the RMSDL parameter for each residue on the temperature and pressure using two-way ANOVA (see Methods). The influence of temperature and pressure on the RMSDL parameter was significant, if the corresponding *F*-statistics was above the threshold at *α* = 0.05 (see Additional file 4).

**Figure 5:**
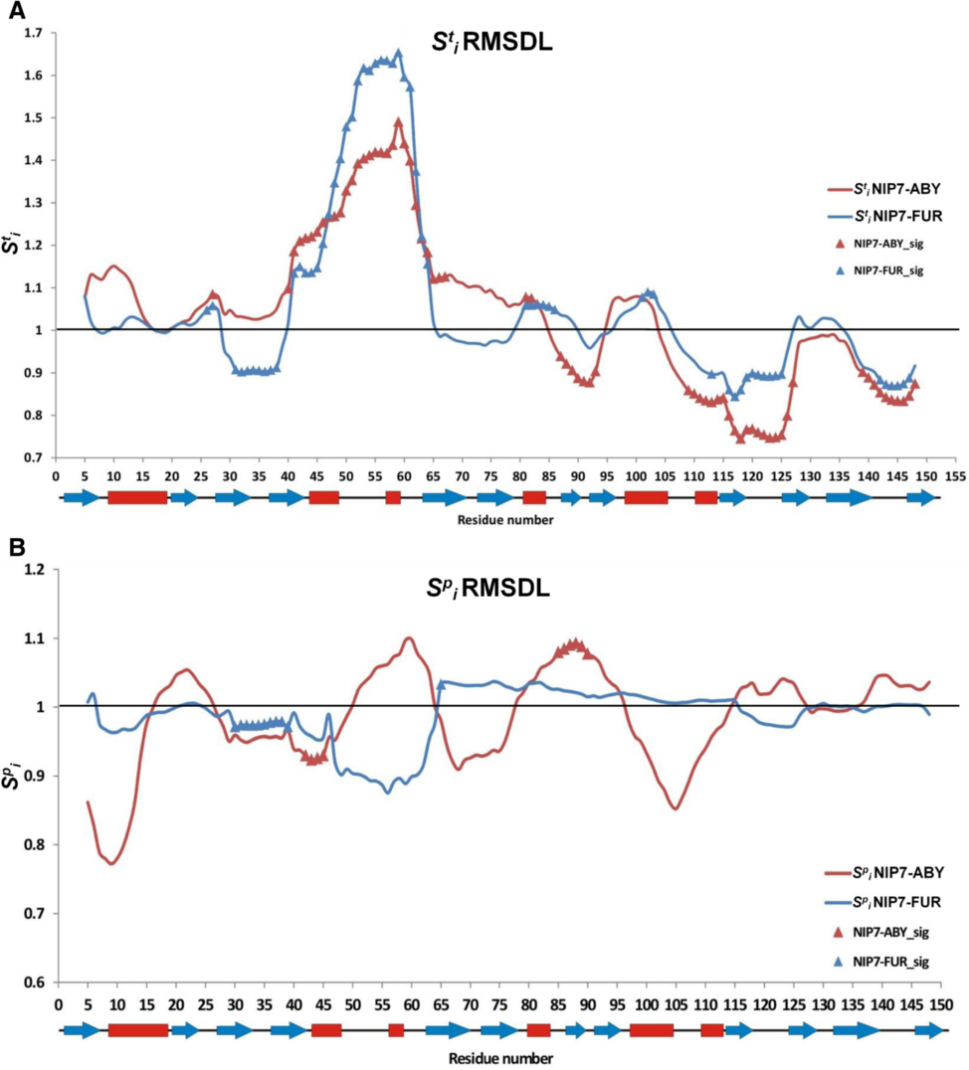
**Graphs showing the dependencies of the RMSDL index of sensitivity to increase in temperature and pressure. (A)** Lines, graphs of the RMSDL index of temperature sensitivity at protein positions during increase in temperature for NIP7-ABY (red) and NIP7-FUR (blue). Triangles highlight the positions at which the RMSDL parameter significantly depends on temperature. **(B)** Lines, graphs of the RMSDL index of pressure sensitivity at protein positions during increase in pressure for NIP7-ABY (red) and NIP7-FUR (blue). Triangles highlight the positions at which the RMSDL parameter significantly depends on pressure.

Figure 5A illustrates that temperature sensitivity of the RMSDL parameter is different, being dependent on protein position in the two models. To begin with, there are protein regions for which temperature significantly increases the RMSDL ($S_{i}^{t}$ >1) for both models. To such regions belongs, in the first place, the long segment extending from the C-terminal part of the *β*_4_ strand to the N-terminal end of the *β*_5_ strand (positions 41–65). The *F*-statistics for most of its residues exceeds by many times the 5% critical value for both models (Additional file 4). In the graphs given in Additional file 3: Figure S1, this region corresponds to the peak RMSDL values, which increase when temperature elevates. A small, yet significant, increase in the RMSDL is established in the N-terminal domain for both models at positions 26–27 (the *β*_3_ strand of the N-terminal domain). A significant increase in the RMSDL residues at positions 31–37 (*β*_3_-*β*_4_) under the influence of high temperature is also characteristic of the NIP7-FUR model. High values of the $S_{i}^{t}$ parameter are observed for the N-terminal regions of both models, however, the influence of temperature on change in the RMSDL for these residues proved to be insignificant.

An interesting pattern was observed for the C-terminal domain. $S_{i}^{t}$ <1 for the great majority of residues, the differences were significant for many of them. This meant that elevation in temperature made the conformation of the C-terminal domain to a great extent similar to that of the crystal structures. These changes were the most prominent for region 109–127 (*α*_6_–*β*_10_). This was associated with lower $S_{i}^{t}$ values characteristic of the NIP7-ABY model. Another region, which was also subject to conformational change at elevated temperature in the models, joined two protein domains (positions 80–93). A significant decrease in the RMSDL was characteristic of the C-terminal residues in both models, too.

The sensitivity of the RMSDL values to elevated pressure is shown in Figure 5B. We compared $S_{i}^{p}$ and $S_{i}^{t}$ with respect to the positions at which their proportions are significantly different for both models. We found that their number is considerably smaller for $S_{i}^{p}$ than $S_{i}^{t}$. For the NIP7-FUR model, significant changes are observed in the N-terminal domain (positions 30–38). For the NIP7-ABY model, fluctuations in the $S_{i}^{p}$ values are more expressed, however, changes at positions 41–44 (a decrease in the RMSDL) and 85–89 (an increase in the RMSDL) are significant.

### 2.4 Analysis of the secondary structure

To describe in more detail the conformational changes, we analyzed the secondary structure of the Nip7 protein models at different MD trajectories. Additional file 3: Figure S2 provides evidence for the stability of the secondary structure during the simulations. For example, although the RMSDL values for the loop 69–79 (the *β*_5_-*β*_6_ region) are high (Additional file 3: Figure S1), the secondary structure pattern of the polypeptide chain demonstrates no changes dependent on pressure and temperature. This may suggest that this *β*-hairpin changes its conformation without breaking hydrogen bonds, just by bending or twisting.

Analysis of the data on the changes in the protein secondary structure (Additional file 3: Figure S2) shows also that a part of its elements is unstable when pressure and temperature change. For example, this is characteristic of the loop formed by residues 48–62 (the *α*_2_-*α*_3_ segment). At different pressures and temperatures, the conformation of these residues varies from the states of the *α*-helix (H), the 3_10_ –helix (G), the turn (T), and to the bend (B). A local maximum of the deviations of the polypeptide chain from the crystal structure, the RMSDL (Additional file 3: Figure S1) are observed in the segment. Thus, this protein segment proves to be very mobile.

Other regions of smaller size with unstable secondary structures are the first N-terminal residues, the *β*_10_ terminal residues (transitions from the loop to the extended conformation) and the *β*_11_–*β*_12_ loop (transitions between the turn, the bend and the loop conformations). These regions show characteristic transitions from the loop to the bend, also the maximum RMSDL values (Additional file 3: Figure S1).

**Figure 6:**
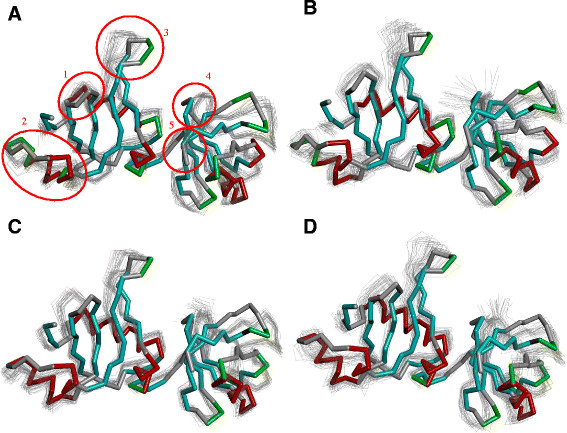
**Representative snapshots of the superposition of the trajectories of protein models with the** ***P. abyssi*** **Nip7 crystal structure. (A)** NIP7-ABY at 300К and 100 MPa; **(B)** NIP7-ABY at 373К and 100 MPa; **(C)** NIP7-FUR at 300К and 100 MPa; **(D)** NIP7-FUR at 373К and 100 MPa. The Nip7 *P. abyssi* crystal structure is in color tube; beta strands, blue; alpha-helices, red; turns, green. Models obtained by the MD simulations are indicated by grey lines. Ovals encircle: (1) the *β*_3_-*β*_4_ loop (residues 29–41), (2) the *α*_2_–*α*_3_ segment (residues 44–61); (3) the *β*_5_-*β*_6_ loop (residues 62–79); (4) the *β*_9_-*β*_10_ loop (residues 120–125); (5) the C-terminal domain (residues 153–155).

The above described conformational changes are supported by the trajectory snapshots providing new dimensions of the credibility of our models (Figure 6; Additional file 3: Figure S3). Thus, residues 69–75, which belong to the *β*-turn, have a stable secondary structure, but high RMSDL values. These residues bend towards the globular part of the N-terminal domain during the simulations. In so doing, they twist slightly the *β*-sheet. The sweep of the loop 69–75 bend depends on pressure and temperature. Similar conformational changes (the bend towards the centre of the N-terminal domain without significant impairment of the secondary structure) are also observed for the loop between the *β*_3_-*β*_4_ strands. It also shows characteristic RMSDL deviations (Additional file 3: Figure S1). As seen in Figure 6B, the range of the fluctuations widens at high temperatures in the NIP7-ABY model, a similar widening is true for the NIP7-FUR model (Figure 6, C, D). The structural fluctuations in the segment 45–55 and those in the loops between the *β*-strands of the terminal domain are conspicuous, too.

### 2.5 Fluctuations in the polypeptide chain

We estimated the structural flexibility of the two proteins at different temperatures and pressures. The graphs display the dependencies of the Root-Mean-Squared-Fluctuation (the RMSF) on the residue number (Additional file 3: Figure S4).

The peak regions of the RMSF values enclose predominantly loops in the sequence regions at positions 31–41 (the *β*_3_–*β*_4_ loop), 50–60 (the *α*_3_ region), 69–79 (the *β*_5_–*β*_6_ region), 98–110 (the *β*_8_-*α*_5_ region), 120–125 (the *β*_9_–*β*_10_ region), 132–135 (the *β*_10_–*β*_11_ region), 140–150 (the *β*_11_–*β*_12_ loop). These regions correspond to those where the RMSFs are the largest. In addition, the highest values for the local root-mean-square deviations of the polypeptide chain (the RMSDLs) are observed in them (compare with Additional file 3: Figure S1).

The other features are the high RMSF values for the residues at the amino (N) and carboxy (C)-termini of the proteins. Notably, the RMSF values are somewhat higher for the trajectories at high temperature than for those at 300 K. These differences were observed for both NIP7-ABY and NIP7-FUR (Additional file 3: Figure S4).

**Figure 7:**
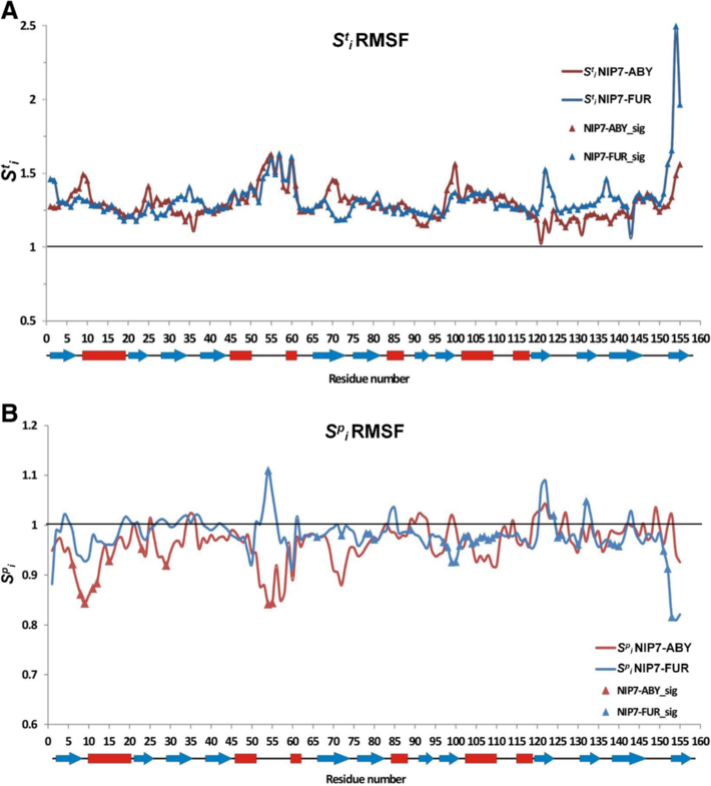
**Graphs showing the dependencies of the RMSF index of sensitivity to increase in temperature and pressure. (A)** Lines, graphs of the RMSF index of temperature sensitivity at protein positions during increase in temperature for NIP7-ABY (red) and NIP7-FUR (blue). Triangles highlight the positions at which the RMSF parameter significantly depends on temperature. **(B)** Lines, graphs of the RMSF index of pressure sensitivity at protein positions during increase in pressure for NIP7-ABY (red) and NIP7-FUR (blue). Triangles highlight the positions at which the RMSDL parameter significantly depends on pressure.

The sensitivity of the RMSF value to increase in temperature and pressure was analyzed for each and every amino acid residue in the same manner as was for the RMSDL parameter. The results are shown in Figure 7 (see Additional file 5 for the two-way ANOVA results). Analysis of the sensitivity profiles of the residues to temperature demonstrated that increase in temperature raised the value of the local polypeptide fluctuations for all the residues at high significance ($S_{i}^{t}$ >1). However, the graph displays regions weakly sensitive to increase in temperature, even insensitive to it (these are residues 36, 121, 123 and 131 for the NIP7-ABY model and residue 146 for the NIP7-FUR model). The RMSF values are high in the residues of the loop already at 300 K, while increase in temperature does not cause any considerable increase in their mobility.

The results are different for fluctuations in the Nip7 models of the deep- and shallow-sea organisms at increased pressure (Figure 7B). The $S_{i}^{p}$ parameter is above unity for a small fraction of residues. For the N-terminal domain, the decrease in fluctuations under the effect of pressure is greater for the NIP7-ABY than the NIP7-FUR model. In this domain, pressure significantly influences residues located at positions 6–15 (the N-terminal part of the *α*_1_ helix), positions 23, 29 (the loop between *β*_2_ and *β*_3_), and 54–55 (the *α*_2_–*α*_3_ loop).

For the NIP7-FUR RMSF, a significant influence of pressure is observed in residues mostly in the C-terminal part of the protein. A wide region of these residues spans positions 97–110 (the *α*_5_ helix), the most sensitive region to increase in pressure and $S_{i}^{p}$ <1 (decrease in the RMSF). Several other residues for which RMSF decreased by pressure significantly are located the *β*_10_ region (positions 125–130), *β*_11_ (138–140), *β*_12_ (152–153) and at position 89. Several positions demonstrating significant decrease in the RMSF under pressure are located in the N-terminal domain (66, 72, 78–80). There are, however, residues for which the RMSF changes significantly when pressure increases. They are observed for NIP7-FUR only (54, 124, 132). Interestingly, the single position, which demonstrates the significant dependence of its RMSF value on the pressure for both models, 54, is located in the *α*_2_–*α*_3_ loop. However, the influence is inversed for this residue in the NIP7-FUR and NIP7-ABY models.

To analyze the influence of pressure and temperature on fluctuations in the models, we performed two-way ANOVA at different temperatures for both the entire models and separately for each domain. The mean RMSF values for the C_α_ atoms in the NIP7-ABY and NIP7-FUR models at different trajectories were compared (see Additional file 6). Statistical analysis demonstrates that, for the NIP7-ABY model at 300 K, change in pressure does not affect significantly fluctuations in the N- and C- domains. However, differences in fluctuations between these domains are significant. In general, the C-terminal domain demonstrates larger fluctuations compared with the N-terminal domain. At high temperature, the influence of pressure on the RMSF of two domains becomes significant. The fluctuations in both domains decreased at high pressure. The difference in the fluctuations between two domains at high temperature remains the same as at 300 K. In other words, the fluctuations are greater for the C-terminal domain.

For the NIP7-FUR model, pressure exerts a significant influence on the RMSF values in the two domains both at low and high temperatures (see Additional file 6). The fluctuations in both domains decrease with increase in pressure, like in the NIP7-ABY model. The differences in the RMSF values for the two domains are significant at 300 and 373 K.

### 2.6 Analysis of salt bridges

The crystal structure of *P. abyssi* contains eight pairs of residues forming salt bridges [28]. It should be noted that the positions of the side chain atoms have not been resolved for some residues capable of forming salt bridges probably due to their decreased stability under crystallization conditions. These are GLU51, LYS76, for example. In our simulations it is feasible to reconstruct side chain atoms for these residues and to take them into account in salt-bridge stability analysis. For each and every possible salt bridge, the proportion of structures was estimated where such a bridge was formed (salt bridge persistence; see Methods). This was done for all the trajectories and models under study. Analysis of the NIP7-ABY and NIP7-FUR models revealed that there were stable salt bridges (occurring, on average, in 70% of the structures in the different trajectories of the two proteins), moderately stable (20–70% persistence) and a number of unstable salt bridges occurring in less than 20% of the structures (Additional file 7). The number of stable and moderately stable salt bridges was larger in NIP7-ABY, twenty, than in NIP7-FUR, thirteen. Interestingly, the residues with unresolved side chain atoms in the crystal structure formed moderately stable salt bridges only in the NIP7-FUR and NIP7-ABY models.

Six salt bridges (GLU10-ARG4, GLU75-ARG37, ARG37-GLU10, LYS20-GLU17, ARG148-ASP109, GLU33-ARG4) are the most stable in the NIP7-ABY model (Additional file 7). These form in more than 70% of the structures in both the NIP7-ABY and NIP7-FUR models. The GLU33-ARG4 and GLU131-ARG116 pairs (unresolved in the 2P38:A crystal structure) may be referred to the most conservative in NIP7-FUR (being moderately stable in NIP7-ABY). Four stable salt bridges from the N-terminal domain (GLU10-ARG4, GLU75-ARG37, ARG37-GLU10, GLU33-ARG4) form a network that links the alpha-helix and the beta-strands. The remaining salt bridges may be assigned to the moderately stable. Some stable or moderately stable salt bridges in NIP7-ABY are unstable in the NIP7-FUR (148–109, 147–113). There are also two salt bridges moderately stable in the NIP7-FUR and unstable in the NIP7-ABY models (146–112, 76–17) (Table 2).

**Table 2:** Sensitivity of the most stable salt bridges in the NIP7-ABY, the NIP7-FUR models to increase in temperatures and pressures

**Salt bridge**	**Marker**	**Function**	**NIP7-ABY**				**NIP7-FUR**			
			***S***^***T***^	***F (T)***	***S***^***p***^	***F (P)***	***S***^***T***^	***F (T)***	***S***^***p***^	***F (P)***
10–4	All	*α*_1_–*α*_1_	0.94	2.74	1.04	0.83	0.99	1	1.01	1
75–37	All	*β*_6_–*β*_4_	1.06	0.52	1.11	0.91	0.99	1.89	1.00	0.36
37–10	All	*β*_4_–*α*_1_	1.04	0.16	1.14	0.99	1.18	2.87	0.88	**4.68**
148–109	All	RNA binding - RNA binding	0.95	0.40	1.09	0.45	u	u	u	u
33–4	All	*β*_3_–*β*_1_	0.92	0.59	0.94	0.69	0.98	0.10	1.00	0.20
20–17	All	*α*_1_–*α*_1_	0.92	2.65	1.02	0.13	0.89	2.96	0.98	0.36
16–12	P.a.&P.f.	*α*_1_–*α*_1_	1.14	3.77	0.91	1.68	1.08	1.60	0.89	2.76
147–113	P.a.&P.f.	RNA binding - RNA binding; *α*_6_	0.98	0.02	0.72	3.18	u	u	u	u
33–2	2p38&P.a.	*β*_3_ - N-terminal	0.98	0.01	0.99	2.07	-	-	-	-
131–116	All	C-terminal - *β*_9_	1.93	**12.23**	0.94	0.49	0.98	0.72	0.99	0.43
51–5	P.a.&P.f.	*α*_2_–*β*_1_	1.25	1.50	1.03	0.03	0.88	1.11	1.18	0.74
76–70	P.a.&P.f.	*β*_6_ - N-terminal	1.20	**6.59**	0.84	**3.42**	0.83	**8.19**	0.95	1.98
147–146	P.a.	RNA binding - C-terminal	1.84	4.12	0.95	0.12	-	-	-	-
48–45	P.a.&P.f.	*α*_2_–*α*_2_	1.06	0.14	0.92	1.42	0.99	0.00	0.88	1.29
88–23	P.a.	*β*_7_–*β*_2_	1.26	**5.91**	0.87	1.72	-	-	-	-
151–109	All	RNA binding; *β*_12_ - RNA binding	0.93	0.09	0.75	1.49	0.86	2.74	0.92	0.42
119–117	P.a.	*β*_9_–*β*_9_	0.98	0.04	1.26	2.31	-	-	-	-
48–44	2p38&P.a.	*α*_2_–*α*_2_	0.99	0.00	0.98	0.01	-	-	-	-
146–112	All	C-terminal - *α*_5_	u	u	u	u	1.01	0.00	1.11	0.50
76–17	P.a.&P.f.	*β*_6_–*α*_1_	u	u	u	u	0.83	1.07	0.95	0.56

Columns: residue pairs forming salt bridges; occurrence of a salt bridge in the different structures (ALL, the NIP7-ABY, the NIP7-FUR models, and the crystal 2P38:A structure; ABY & FUR for only the NIP7-ABY, NIP7-FUR models and so on); the values of the indices of sensitivity for the NIP7-ABY; for the NIP7-FUR models; the F-value for a concrete parameter. Values expressing significant changes, maintenance of a salt bridge when pressure or temperature changes are in bold. Unstable salt bridges (occurrence less than 20%) are marked with the symbol u.

Analysis of the functional role of the most stable salt bridges demonstrated (Table 2) that most of them stabilized the packaging of the elements of the protein secondary structure. However, a number of salt bridges in our models formed residues for which interactions with RNA has been demonstrated [23]. There are four such pairs among stable bridges for the NIP7-ABY model and only one for NIP7-FUR model (148–109) is formed by a residue pair with both involved in the interaction with RNA, the other salt bridges are either unstable or missing in the NIP7-FUR model.

How high temperatures and pressures may affect the formation of salt bridges in the two different protein models? To answer this question, we introduced for each salt bridge its index of sensitivity to pressure (*S*^*p*^) and temperature (*S*^*t*^) in the same manner as we did for the RMSDL and the RMSF parameters (Methods). The higher were the values of these indices, the greater was the persistence of a bridge at high pressure or temperature. To estimate the significance of the influence of pressure and temperature on the stability of a salt bridge, we applied two-way ANOVA and obtained values of the statistics *F*(T), *F*(P) (see Methods). We assumed that high temperature or pressure exerted an influence on stability of a salt bridge, if the corresponding *F*(T) and *F*(P) statistics exceeded the 0.05 significance threshold. The results for the stable and moderately stable salt bridges common to both models are given in Table 2, Additional files 8 and 9. They demonstrate that for the NIP7-ABY model, of the 20 stable salt bridges 3 (15%) are affected by high temperatures (131–116, 76–70 and 88–23). One salt bridge is significantly subject to the influence of pressure (76–70) both also become stable at high temperature. This bridge becomes also stable at high temperatures in NIP7-FUR. Both models also contain salt bridges, whose temperature sensitivity considerably, yet insignificantly, deviates from 1 (*S*^*t*^ <0.8 or *S*^*t*^ >1.2). Most become stable when temperature rises (*S*^*t*^ >1). Thus, elevation in temperature for a number of salt bridges in our models changes their persistence mainly in favor of its increase.

A more detailed consideration of the salt bridge stability may reveal certain interesting details. Bridge 131–116, whose stability, in the case of the deep-water protein, is under the significant influence of temperature, and is formed by the side chain groups of residues ASP131 and ARG116 for the NIP7-ABY model. In the case of the shallow-water protein, aspartic acid is substituted by glutamic acid at position 131 and no significant influence of temperature is observed. Bridge 148–109 is formed by the side chain groups of residues ARG148-ASP109 for the NIP7-ABY model. In the case of the shallow-water protein, arginine is substituted by lysine at position 148 and, as a result, this bridge turns out to be unstable for the NIP7-FUR model. Therefore, the structure of the side chain radicals has a strong impact on the stability of these salt bridges.

### 2.7 Influence of protein type on the dynamics of protein structure

To estimate the influence amino acid substitutions exerted on the structural properties of the NIP7 proteins in comparison with factors such as temperature and pressure, we performed three-way ANOVA for each residue. The results are given in Additional file 10.

**Figure 8:**
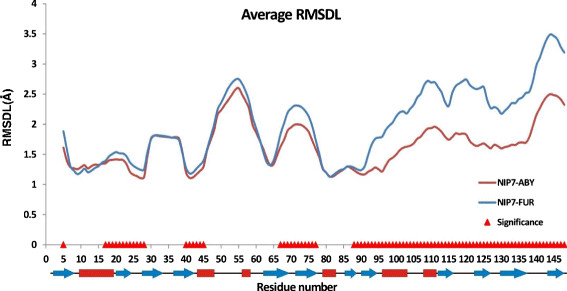
**The RMSDL values for the NIP7-ABY and NIP7-FUR proteins averaged for all the trajectories and runs.** Red triangles highlight the positions at which the RMSDL parameter significantly depends on model type.

Analysis of the influence temperature exerts on the RMSDL in the two models shows that the local structure deviates significantly from the crystal 2P38:A for 77 of the 143 positions we analyzed (53%). Pressure exerts a significant influence on the RMSDL at only 9 positions of the protein (6%), while protein type exerts a significant influence on the conformational deviations of the polypeptide chain for 91 positions, 63% (Figure 8).

The most characteristic differences are observed for the C-terminal domain of the protein (Figure 8). Furthermore, significant differences in the RMSDL parameter depending on model type are observed for regions 16–28, 40–45, 67–77 and position 5. As seen, the deviations from the crystal structure are greater for the NIP7-FUR than the NIP7-ABY model.

A similar analysis was carried out for the RMSF parameters of the amino acid residues (Additional file 10). The results showed that a rise in temperature caused a significant increase in the RMSF for all, without exception, amino acid residues of the NIP7 protein. Pressure exerted a significant influence on change in 26 amino acid residues (16%). Type of model exerted an influence on change in fluctuations for 56 (36%) of the amino acid residues.

**Figure 9:**
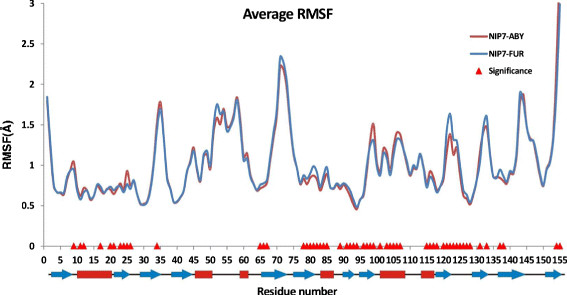
**The RMSF values for the NIP7-ABY and NIP7-FUR protein averaged for all trajectories and runs.** Red triangles highlight the positions at which the RMSF parameter significantly depends on model type.

Significant differences between the RMSF values for the residues of the two models were observed both in the case when fluctuations were higher for the NIP7-ABY model and, conversely, for the NIP7-FUR model (Figure 9). Residues at positions of the N-terminal domain (9, 11, 12, 17, 20, 21, 23, 24–26) corresponding to the *α*_1_ helix and the *β*_2_ strand, also position 34 (the loop between *β*_3_ and *β*_4_), also the C-terminal domain (96–107, 115–118, 154–155) are referred to the first group. Positions 65–67, 78–85, 89, 120–128, 131–133, 138–139 are referred to the second group.

## 3 Discussion

### 3.1 Effect of temperature and pressure on the structure and dynamics of Nip7

In our work we compare the dynamics of two Nip7 protein models, NIP7-ABY from *P. abyssi*, a deep water organism, and NIP7-FUR, from *P. furiosus*, a shallow water organism, at different temperatures and pressures.

The hallmark feature of our models is their stability throughout the simulation period (40 ns). It is well maintained at both elevated temperatures and pressures. The RMSDs from the crystal structure (2P38:A) are, on average, smaller for the NIP7-ABY than the NIP7-FUR model. It could be remembered, however, that the NIP7-FUR model is not crystallographic, but it has been rather produced by homology modeling, which could indeed justify a larger RMSD. The secondary structure of the model changes slightly, thereby providing evidence that the structural differences between the two proteins are related to change in loop conformation and in mutual disposition of the secondary structure elements.

Exposure to high temperature for the two proteins results in change in the equilibrated conformations of the polypeptide chain, on the one hand (seen when comparing the RMSDL profiles in Figure 5), and in increase in its fluctuations, on the other hand.

Conformational changes at increasing temperature are related to decrease in structural deviations from the crystal structure in the C-terminal domain, including the positions that form the interaction with RNA. The deviations increase in the regions of the helices *α*_2_-*α*_3_ in the N-terminal domain. It should be noted that the specific influence of elevated temperature on the conformation of the protein, on its active centers in particular, is one of the possible mechanisms providing optimal protein activity in this region. For example, the important role of temperature-dependent conformational transitions of protein, at its active site particularly, in the enzymatic activity of the NADH oxidase from *Thermus thermophilus* has been well demonstrated at high temperatures [29]. Also it was demonstrated that some mutation may increase and some of them may decrease the thermostability of thioredoxins from *Escherichia coli* and *Bacillus acidocaldarius*; the thermostability of these proteins was revealed to depend on ionic interactions between the thermolabile regions [30]. Such conformational changes may be important to the Nip7 functions as well.

The comparison of the protein models obtained at 373 and 300 K demonstrates that at high temperature the fluctuations in the polypeptide chain increase, although the compactness of the protein structure is preserved (Figure 3). The increase in the fluctuations of the polypeptide chain is non-uniform. The regions subject to the fluctuations are mainly loops and the terminal regions of the protein. Like in the case of the RMSDL, the region *α*_2_-*α*_3_ proved to be the most subject to increase in the RMSF among the internal regions of the chain. It is pertinent to note that a similar effect of high temperatures on the conformation of the polypeptide chain is characteristic of the high temperature protein models [31]-[34].

The effect of increased pressure on protein structure is manifold. The structural deviations of the NIP7-ABY from the conformation of the Nip7 model based on the X-ray diffraction structural data at high temperatures decrease as pressure increases. This trend holds true for the NIP7-FUR model (Table 1). The increased pressure results in a decrease in the accessible surface area. This decrease is characteristic of NIP7-FUR for which the influence pressure exerts on the SAS value is significant (Additional file 2). A significant decrease in the SAS area is observed for the NIP7-ABY model, only at high temperature. In this way, increase in pressure makes the Nip7 structure more compact, an effect more expressed at high temperature.

The increase in pressure produces structural rearrangements of the protein. Thus, for the NIP7-ABY model, this increase results in a decrease in the local deviations of protein conformation from the crystal structure. This is in complete agreement with the conclusions that the pressure affects essentially slower motions which imply structural rearrangements of the protein globule [35]. The pressurization effect is more pronounced for the NIP7-ABY model and it affects the conformation non-uniformly, increasing the RMSDL in some regions and decreasing it in others (Figure 5B). As for the NIP7-FUR model most conformational changes occur under high pressure in the N-terminal domain.

A decrease in the fluctuations in the polypeptide is another effect of an increase in pressure we observed both at the domain (Additional file 6) and the residue levels (Figure 7B).

The MD simulations of protein structures subject to increased pressure have yielded abundant evidence of reduced fluctuations in the polypeptide chain due to pressurization [35]-[38]. They are convergent in demonstrating that, if the protein globule does not denaturate, pressure can stabilize the polypeptide chain. An increase in protein stability resulting from an increase in pressure has been demonstrated experimentally for the exemplary glutamate dehydrogenases from the hyperthermophilic archaea *Pyrococcus furiosus*[39] and *Thermococcus litoralis*[40]. The published data support ours: indeed, an increase in pressure stabilized significantly the protein globule, also the protein complex formed by the enzymes. The authors suggested that such a stabilization may arise through changes in the stability of the native states due to reduced fluctuations in the polypeptide chain at high pressures and temperatures [39]. However, our results demonstrate that the magnitude of the effect can be different for protein parts and for proteins from different organisms.

### 3.2 Functional implications for Nip7

Our current results demonstrate that the C-terminal domain is subject to larger structural displacements and fluctuations than the N-terminal domain during the MD simulations. This domain is plastic possibly because it is small, with just ~60 amino acid residues, and is stabilized predominantly by the hydrophobic core (the few salt bridges in this domain coordinate the positions of the loops and the terminal helical regions). The plasticity of the DNA/RNA-binding domains makes non-specific nucleotide-binding feasible [41]-[43]. Such a flexible structure, in contrast to the strictly coordinated components, which obey the key-lock rule, provides the possible binding to the poly-U RNA and poly-AU RNA sequences with a weak secondary structure [22],[23]. Therefore, the plasticity that we currently observed for the Nip7 PUA-domain may presumably be its significant functional property.

The Nip7 PUA-domain has an interesting feature: the formation of salt bridges by the residues shaping the interaction with RNA. Possibly, this effect allows the conformation of chain side groups of these residues to stabilize so as to facilitate binding to RNA. It is encouraging that a similar effect of the stabilization of the side group amino acids at the expense of the formation of rigidifying salt bridges has been observed in the active centre of acylphosphatase from *Pyrococcus horikoshii*[44]. This is pertinent to our current observations: such a mechanism is to a great extent characteristic of the model for the deep-water NIP7-ABY protein.

The N-terminal domain, on the whole, is subject to smaller fluctuations compared to the C-terminal domain (Additional file 6). In this connection, the structurally unstable segments of this domain (positions 30–40, 49–59, 69–79) are outstanding. Two of these segments are *β*-hairpins (positions 30–40, 69–79), the deviations from the crystal structure in these segments occur without significant changes in the secondary structure, so that they bend in the direction of the *α*_1_-helix in the two models. These conformational changes may result from interactions in the salt bridges network (GLU10-ARG4, ARG37-GLU10, GLU75-ARG37) bringing close together the *β*-sheet and the *α*_1_ helix of the N-terminal domain. This interaction network may, in turn, become stabilized through the voluminous hydrophobic nucleus formed by hydrophobic residues in the centre of the *β*_1_-*β*_5_ strands.

In terms of the putative functional role in the N-terminal domain, it appears worthwhile to consider the loop between the second and the third *α*-helices (positions 49–59). The loop is contiguous with a lengthy nonpolar region at the surface of the N-terminal domain, which extends from the *α*_2_ helix to the groove between the N- and C-terminal domains so that is comes to lie on the side opposite to the polar portion of the Nip7 surface (Figure 1) [23]. The conformational changes in the *α*_2_-*α*_3_ region may be due to the high twisting tension of the main chain in it. It undergoes a sharp bend at the very end of the short *α*_3_-helix, then forms one helix turn, which is converted into the *β*_5_ strand structure (Figure 1A). The positions of the N- and C- terminal residues of this fragment are fixed by the globule of the N-terminal domain. Then, the loop itself comes to lie aside from the globular domain without imposing steric constraints on the conformational changes. Additional destabilizing factors for the conformation of this region are alternating polar and hydrophobic (positions 46, 49, 53, 55) residues, which, as a result of conformational changes in the main chain of the *α*_2_-*α*_3_ segment, can by turns face solvent. It may be assumed that this bend together with the lengthy region of the nonpolar surface may play an important role in the interaction of the Nip7 protein with the exosomal protein partner [20],[22]. Interestingly, prediction of the protein-protein interaction sites using 3D structure by the SPPIDER web-server demonstrated that most residues of this region could be involved in protein-protein interactions with high probability both for the NIP7-ABY and NIP7-FUR models (Additional file 11).

The structural lability of proteins may accomplish a role of consequence in molecular recognition. For example, the regions of the polypeptide chain of the monomers devoid of an orderly structure can provide the protein interaction [45] and adopt an orderly conformation in the process of binding [46]. With respect to the Nip7 protein, intrinsic flexibility appears to be more likely [47] because it ensures the presence of a conformational ensemble required for the formation of RNA-protein and protein-protein interactions [48].

### 3.3 Comparison of the protein dynamics between the shallow- and deep-water organisms

The sequences of *P. abyssi* and *P. furiosus* Nip7 in the 3D structure differ by 47 substitutions (~30%; Figure 1B); 19 of them result in radical changes of the physico-chemical properties of amino acids. These substitutions may be assigned to categories I-V.

(I) Substitutions resulting in the replacement of a polar residue in *P. abyssi* by a nonpolar in *P. furiosus* (7 substitutions): 2 (R → I), 21 (Y → F), 58 (Y → F), 59 (S → A), 66 (T → M), 80 (N → A), 119 (K → V).

(II) * P. abyssi* → *P. furiosus* substitutions, which result in replacement of a charged amino acid by an uncharged one at retained polarity (5 substitutions): 23 (E → T), 27 (E → N), 44 (E → N), 144 (K → S), 154 (K → T).

(III) Substitutions of nonpolar amino acids in *P. abyssi* by polar ones in *P. furiosus* (1 substitution): 140 (L → R).

(IV) * P. abyssi* → *P. furiosus* substitutions of the uncharged polar side group by the charged side group: 121 (Q → K).

(V) Substitutions *P. abyssi* → *P. furiosus* resulting in the change of the charge sign to the opposite one (5 substitutions): 88 (K → D), 113 (E → K), 117 (K → Q), 147 (R → E).

As a result of such substitutions, the hydrophobic portion of SAS increased in the shallow-water organism compared with its deep-water counterpart. The question then was: How could this influence the stability, dynamics and functional properties of the proteins? True, both proteins, in all the conditions we examined, proved to be stable and had a similar conformation. However it was perplexing to single out a parameter in the dynamics that could be significantly influenced by these substitutions.

**Figure 10:**
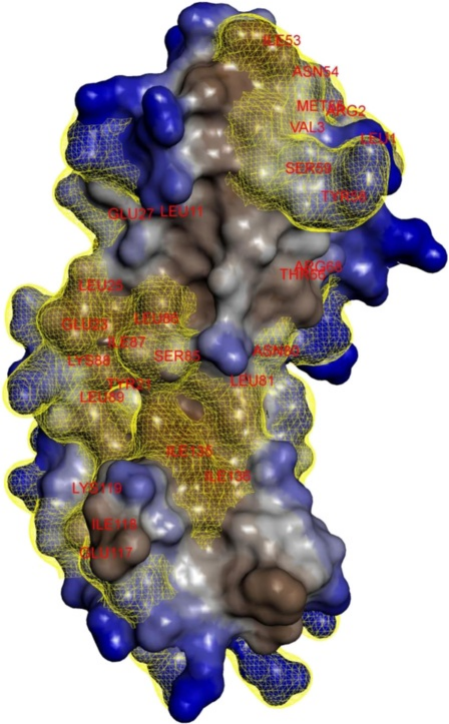
**Surface localization of the Nip7 residues substituted in** ***P. abyssi*** **→** ***P. furiosus*.** The surface of the *P. abyssi* Nip7 [23] (2P38:A) was built using the Accelrys Discovery Studio Visualizer with a 1.4 Å radius of water molecule. Surface region: polar, blue; hydrophobic, brown. Yellow color denotes substituted residues.

It will be remembered that the majority of substitutions that belong to categories I and II lie in the N-terminal domain of the protein, they are in contact with its surface region, which contains mobile residues (the *β*_2_-strand and the *β*_2_-*β*_3_ loop), also residues at positions *α*_2_-*α*_3_ (Figure 8) and it may presumably be involved in protein-protein interactions (Figure 10; Additional file 11).

The substitutions we observed in the N-terminal domain may, probably, somehow modify the interaction character of this region with its molecular partner. Substitutions in this stretch are associated with increase in hydrophobicity of residues in the shallow-water organisms. Changes of such kind can promote the stabilization of protein interaction of Nip7 and its partner. This is because hydrophobic interactions contribute considerably to the stability of protein-protein interfaces [49]. Another fact will be remembered here. When pressure close to atmospheric and temperature rises, hydrophobic interactions favor closer interactions of nonpolar protein regions [50], which can additionally stabilize the Nip7-exosome protein complex in *P. furiosus*. If so, the observed substitutions may truly be of adaptive character. We have previously analyzed [51] the indices of asymmetry for amino acid substitutions in hundreds of *Pyrococcus* proteins starting from its common deep-sea ancestor *P. furiosus*, *P. horikoshii* and *P. abyssi* to the extant shallow-sea organism *P. furiosus*. According to the results, the values of the indices are positively correlated with the scales of amino acid hydrophobicity and negatively with the polarity scales indicating that polar residues were predominantly substituted by nonpolar when the shallow-sea habitats replaced the deep-sea ones. However, the issue as to whether this trend is a reflection of the stabilization of protein-protein interactions or of the entire structure requires further study.

Furthermore, the character of changes in fluctuations for the chain stretch in the *α*_2_-*α*_3_ region at increasing pressure is drastically different for the two proteins: they significantly increase in the shallow-water protein and decrease, conversely, in its deep-water counterpart (Figure 7А). A general decrease in fluctuations throughout the entire N-terminal domain at increasing pressure is also characteristic of the deep-sea protein. The magnitude of fluctuations for efficient binding must be within definite limits and their general decrease at reduction in pressure is also additive in the deep-water protein.

Substitutions exert a significant influence on the conformational parameters in proteins connecting two protein domains (the RMSDL and the RMSF parameters) in the *β*_6_-*α*_4_ region. This is the stretch that joins together two protein domains. Probably, mutations in it favor change in the mutual orientation of the domains and result in an increase in the structural deviations in the C-terminal domain of the protein in the NIP7-FUR as compared with the NIP7-ABY model (Figure 8).

As for the RNA-binding domain, radical changes that could alter residue charge are characteristic of the shallow-water protein. As a consequence, the formation of certain salt bridges, those between residues involved in RNA recognition in particular, is impaired. It is also worth to note, that the number of residues associated with significant differences in RMSDL/RMSF parameters between the shallow- and deep-water protein models are higher in the RNA-binding domain. The differences we observed in the course of MD simulations may reflect the different ways and means RNA interacts with the Nip7 proteins at high and low pressures.

## 4 Conclusions

The MD simulations we performed currently for the Nip7 protein from the shallow-water (*P. furiosus*) and deep-water (*P. abyssi*) marine hyperthermophylic archaea demonstrated that their structures are stable at different temperatures (300 and 373 K) and pressures (0.1–100 MPa). Increase in temperatures and pressures caused conformational changes in about the same protein regions, in the loops first of all. In the examined ranges of temperatures and pressures, increase in temperature had a more pronounced effect on changes in the dynamic properties of the protein globule than increase in pressure. A number of highly mobile segments of protein globule presumably involved in protein-protein interactions were identified. Substitutions of the polar residues in the deep-water organisms by hydrophobic in the shallow-water ones were observed in some of the regions. These replacements may be evidence of change in the interaction pattern of the Nip7 protein and the proteins of the exosome complex in the course of the divergence of the shallow-water organisms from the deep-water ones. Taken together, the results of such a complex comparative analysis of the MD of proteins from organisms belonging to different ecological niches may be useful in refining ideas of their functioning and adaptation to extreme environmental conditions.

## 5 Methods

### 5.1 Protein models

The model of the *P. abyssi* Nip7 protein was retrieved from the PDB [52]*,* identifier 2P38 [23]. The atomic model of the *P. abyssi* Nip7 contains two independent monomers and 252 water molecules. The monomers are symmetric with respect to each other, with a head-to-tail orientation, they form a dimer without showing structural differences (the RMSD of the C_α_ atoms between two monomers is 0.11 Å). The contact surface between the monomers is about 700 Å^2^ per molecule, or 8% of its total surface. These estimates suggest that the dimeric structure might have resulted from crystallization and, if so, it may be biologically irrelevant [23]. Hence, 2P38:A is the only monomer included in analysis. The first four and the last seven amino acid residues not located in the experiment were discarded. As a result, the total length of the *P. abyssi* Nip7 protein model is 155 amino acid residues. This model is referred to as NIP7-ABY, the amino acid residues are numbered according to their order numbers in the structure (1VAL-155ASP).

The *P. furiosus* Nip7 model (NIP7-FUR) was built using the method of reconstruction based on homology. It was retrieved from the ModBase databank [53], ID Q8TZP7. The *P. abyssi* and *P. furiosus* Nip7 alignments contain neither deletions nor insertions and, hence, NIP7-FUR reliability is ensured (Figure 1B). The number of residues of the NIP7-FUR model is 155, their numbering corresponds to the one for NIP7-ABY (1ILE-155ASP). Based on the C_α_ atoms, the RMSD of the initial structures of NIP7-FUR and NIP7-ABY is 0.1 Å.

Here we examine the MD simulations of the Nip7 protein models at various pressures (0.1, 50, and 100 MPa) and at temperatures 300 and 373 K.

### 5.2 Preparation of models for MD simulation

The MD of the protein 3D structure is simulated using the program package GROMACS [54] 4.5.3. The algorithm for preparation of the models is as follows. To remove undesirable tensions, the structure energy is minimized in vacuum. Then, the molecule is placed in a cubic cell (edge 200 Å) containing water molecules in the SPCE model. Then, ion molecules are added to the cell to neutralize the charge. The NIP7-ABY model consists of 2 585 protein atoms, 32 900 water molecules, and 1 ion. The NIP7-FUR model is composed of 2 544 protein atoms, 32 900 water molecules, and 2 ions. Thereafter, the protein solvent system is minimized for 2 000 steps. There are two different techniques for further preparation of the models. In the case of simulation at 300 K, there are two preparatory steps of simulation at the given pressure-temperature values, 200 ps each; in that of simulation at 373 K, the number of preparatory simulations is nine, with a gradual increase in temperature by 8 K at each simulation. Pressure in the system is set at simulation onset.

### 5.3 MD simulations

MD simulations are performed using the LINCS [55] algorithm in the amber99sb-ildn force field [56]. Electrostatic interactions are computed using the Partial Mesh Ewald (PME) algorithm. Pressure coupling was done using Parrinello-Rahman method [57]. Temperature coupling was done using v-rescale method [58].

The simulation time was 40 ns. To take into account slower dynamics of proteins at high pressure due to increase in viscosity of water for each set of parameters we perform 5 independent trajectory runs. As a result, 30 experiments are considered for each protein model.

Data were saved every 80 ps. We collected statistics for protein conformation parameters for model structures after equilibration from 20 to 40 ns.

### 5.4 Structure similarity analysis

We characterized changes in the structures by the C_α_ atoms RMSDs and the solvent accessible surface area of the residues (the SAS); the secondary structure parameters based on the DSSP algorithm [59] were calculated using the GROMACS package with the help of the g_rms, g_sas and do_dssp programs, respectively.

To define the regions of the protein structure most susceptible to pressure/temperature impact, in a sliding window of the size *q + 1,* the local deviation of the structure for each residue, the RMSDL after superposition of two protein structures using all С_α_ atoms was calculated as:

$$\mathrm{RMSD}L_{i}\left(v,w,q\right)=\sqrt{\left(\frac{1}{q}\sum_{j=i-q/2}^{j=i+q/2}{\left(v_{jx}-w_{jx}\right)}^{2}+{\left(v_{jy}-w_{jy}\right)}^{2}+{\left(v_{jz}-w_{jz}\right)}^{2}\right)}\text{,}$$

where **v**, **w** are two structures superposed to minimize their C_α_ RMSD; *i* is the index of residue for which RMSDL is calculated; *v*_*jk*_, *w*_*jk*_ (*k* = *x*,*y*,*z*) are x, y, z coordinates of the C_α_ atoms of the aligned residues *j* in each of the **v**, **w** structures; the *q* parameter was set to 10 yielding a window of 11 amino acids to calculate the RMSDL.

### 5.5 Secondary structure analysis

To analyze the stability of the secondary structure during the simulations, we analyze the 3D model at each step using the DSSP program [59]. A hundred 3D structures obtained at the last steps of the MD simulation were used as input for the DSSP program. The occurrence frequencies of a secondary structure of a particular type at a concrete position in the secondary structure were determined. The total secondary structure for a given trajectory included the type of the secondary structure with the highest occurrence frequency. The procedure was performed on all the 24 trajectories.

To estimate the variability in the secondary structure types in the 12 trajectories for each of two models we used the Protein Variability Server [60].

### 5.6 Analysis of the fluctuation in the polypeptide chain

Fluctuations in the polypeptide chain were characterized by the С_α_ atom RMSFs computed using the GROMACS package program g_rmsf. The average С_α_ atoms RMSF value in Angstroms was computed for all the residues in the Nip7 model and N- and C- terminal domains. To determine the significance of the differences between the average С_α_ atoms RMSFs of the different trajectories or models, we used two-way ANOVA (see below).

### 5.7 Dependence of the models’ conformational parameters on temperature, pressure and protein sequence

To determine the influence of temperature, pressure and amino acid substitutions on the protein parameters, we used ANOVA of two types [61].

First, to estimate the influence of pressure and temperature on the protein structural characteristics (for example, Rg, SAS, salt bridge persistence or RMSF of the *i*-th residue) we used two-way ANOVA. In this analysis the influence of two factors was examined: temperature (T = 300, 373 K) and pressure (P = 0.1, 50, 100 МPа). In each set of modeling parameters, we estimated the means of this characteristic on the basis of the results of analysis of 5 runs. In this way, for the structural characteristic we analyzed the ANOVA table with *r* = 2 (temperature), *c* = 3 (pressure), the same number of observations for each parameter set, whose sum total was *n* = 30 (2 · 3 · 5). We tested the hypothesis that the means of the characteristic are independent of temperature and pressure. The critical *F*-values were set at *α* = 0.05 for *F* (P) = 3.4 (*df* = 2), temperature *F* (T) = 4.26 (*df* = 1), and for interaction of these factors *F* (PxT) = 3.4 (*df* = 24).

Second, to estimate the effect of increased temperature on the conformational parameters of the protein, their index of sensitivity (*S*^*t*^) was computed. To this end, we calculated the average value of a model’s conformational parameter, *X* (for example, the RMSF of the *i*-th residue), for the trajectories obtained at high temperatures (*X*_*T=373*_) and for those obtained at low temperatures (*X*_*T=300*_). The index of sensitivity was expressed as $S^{t}=\frac{X_{T=373}}{X_{T=300}}$. The *S*^*t*^ value close to unity meant that increased temperature had no considerable effect on the value of the conformational parameter compared to its value at low temperature. The *S*^*t*^ value considerably larger than unity implied that increased temperature increased the value of the structural parameter compared to its value at low temperature. The *S*^*t*^ value considerably smaller than unity imply that increase in temperature reduces the value of the structural parameter. A number of conformational parameters, such as the RMSDL_*i*_, the local structural deviations of the polypeptide chain for the *i-*th residue, the RMSF_*i*_, the root-mean-square fluctuations in the *i-*th residue, and salt bridge persistence (see below) were considered.

Similarly, the sensitivity of the structural parameters to increased pressure *S*^*p*^ was estimated. We determined the value of the parameters for the trajectories at high pressures (50 and 100 МPа), *X*_*P-нigh*_, atmospheric and moderate pressure (0.1 МPа), *X*_*P-low*_. The sensitivity to pressure was expressed as $S^{p}=\frac{X_{P-\mathrm{high}}}{X_{P-\mathrm{low}}}$.

Third, in analysis of the structural characteristics of the amino acid residues the RMSDL, the RMSF, we estimated the influence of protein type (NIP7-ABY, NIP7-FUR); in such a way, we treated the three-way ANOVA model that considered temperature, pressure and protein type (*r* = 2, *c* = 3, *l* = 2; *n* = 60). The additional factor (protein type) reflected the influence amino acid substitutions exerted on the RMSDL and RMSF parameters for each of the residues. It should be stipulated, however, that such an analysis did not provide information about the influence of specific amino acid substitutions on changes in a conformational parameter of a residue. The critical *F*-values in this analysis were set at *α* = 0.05 for pressure *F* (P) = 3.4 (*df* = 2), for temperature *F* (T) = 4.26 (*df* = 1), and for interaction of these factors *F* (PxT) = 3.4 (*df* = 24).

### 5.8 Analysis of salt bridges

To identify salt bridges, we used the criterion according to which the distance between two oppositely changed atoms should not exceed 4 Å [28]. Salt bridge persistence was expressed as the proportion of structures in the equilibrium region of trajectories (20–40 ns) for which the salt bridge was identified.

The sensitivity of the salt bridge to temperature (*S*^*t*^) and pressure (*S*^*p*^) was defined as the ration of the salt bridge persistence at high and low temperature and high and low pressure. The *S*^*t*^ (*S*^*p*^) value close to unity meant that increased temperature (pressure) had no considerable effect on the persistence of the salt bridge. The *S*^*t*^ (*S*^*p*^) value considerably larger than unity implied that increased temperature (pressure) increased the persistence of the salt bridge compared to its value at low temperature. The *S*^*t*^ (*S*^*p*^) value considerably smaller than unity imply that increase in temperature (pressure) reduces the persistence of the salt bridge.

The dependence of salt bridge persistence on temperature and pressure was estimated using two-way ANOVA (see above).

### 5.9 Prediction of the protein-protein interactions

To predict the sites of protein-protein interactions, the SPPIDER server (http://sppider.cchmc.org/) was used [62].

### 5.10 Structure visualization

The Accelrys Discovery Studio Visualizer was applied to visualize the structure of the models (http://accelrys.com/products/discovery-studio/).

### 5.11 Statistical analysis

Statistical analysis of the data was performed using the Excel and Statistica 6.0 package.

### 5.12 Availability of supporting data

All the supporting data are included as additional files.

## Abbreviations

MD: Molecular dynamics

Rg: Radius of gyration

RMSD: Root mean squared deviation

RMSF: Root mean squared fluctuation

RMSDL: Local root mean squared deviation

SAS: Solvent accessible surface

NIP7-ABY: Model of the Nip7 protein of *Pyrococcus abyssi*

NIP7-FUR: Model of the Nip7 protein of *Pyrococcus furiosus*

## Competing interests

The authors declare that they have no competing interests.

## Authors’ contributions

KEM and NAA performed the MD simulations of the Nip7 proteins. KEM, DAA and YNV performed the analysis of the MD results. EVB, NAK and DAA initiated the comparative molecular dynamics study of the Nip7 proteins and participated in its coordination. All authors read and approved the final manuscript.

## Additional files

## Supplementary Material

Additional file 1: 2-way ANOVA of change in the radius of gyration, the NIP7-ABY and NIP7-FUR models. Click here for file

Additional file 2: 2-way ANOVA of change in the SAS parameter, the NIP7-ABY and NIP7-FUR models. Click here for file

Additional file 3: **Analysis of the RMSDL, the NIP7-ABY and NIP7-FUR models; Analysis of the secondary structure, the NIP7-ABY and NIP7-FUR models; Snapshots of the NIP7-ABY and NIP7-FUR models at different temperatures and pressures; Analysis of the C**_**α**_**RMSF, the NIP7-ABY and NIP7-FUR models.** Click here for file

Additional file 4: 2-way ANOVA of RMSDL values of each residue, the NIP7-ABY and NIP7-FUR models. Click here for file

Additional file 5: 2-way ANOVA of RMSF values of each residue, the NIP7-ABY and NIP7-FUR models. Click here for file

Additional file 6: 2-way ANOVA of the fluctuations in the C- and N-terminal domains of the NIP7-ABY and NIP7-FUR models. Click here for file

Additional file 7: Analysis of salt bridge persistence during molecular dynamics simulation at different pressures and temperatures. Click here for file

Additional file 8: 2-way ANOVA of salt bridges persistence, the NIP7-ABY model. Click here for file

Additional file 9: 2-way ANOVA of salt bridges persistence, the NIP7-FUR model. Click here for file

Additional file 10: 3-way ANOVA of RMSDL and RMSF fluctuations, the NIP7-ABY and NIP7-FUR models. Click here for file

Additional file 11: Prediction of the protein-protein interaction sites for the NIP7-ABY and NIP7-FUR models by the SPPIDER web-server. Click here for file
