# Supplementary material for: Molecular dynamics simulations of the Nip7 proteins from the marine deep- and shallow-water Pyrococcus species
Source: BMC Struct Biol. 2014 Oct 15;14:23. doi: 10.1186/s12900-014-0023-z (PMC4209456; doi:10.1186/s12900-014-0023-z)
Supplement: Additional file 3: — Analysis of the RMSDL, the NIP7-ABY and NIP7-FUR models; Analysis of the secondary structure, the NIP7-ABY and NIP7-FUR models; Snapshots of the NIP7-ABY and NIP7-FUR models at different temperatures and pressures; Analysis of the C α RMSF, the NIP7-ABY and NIP7-FUR models. [file s12900-014-0023-z-S3.pdf]

Additional File 3.

A **B**

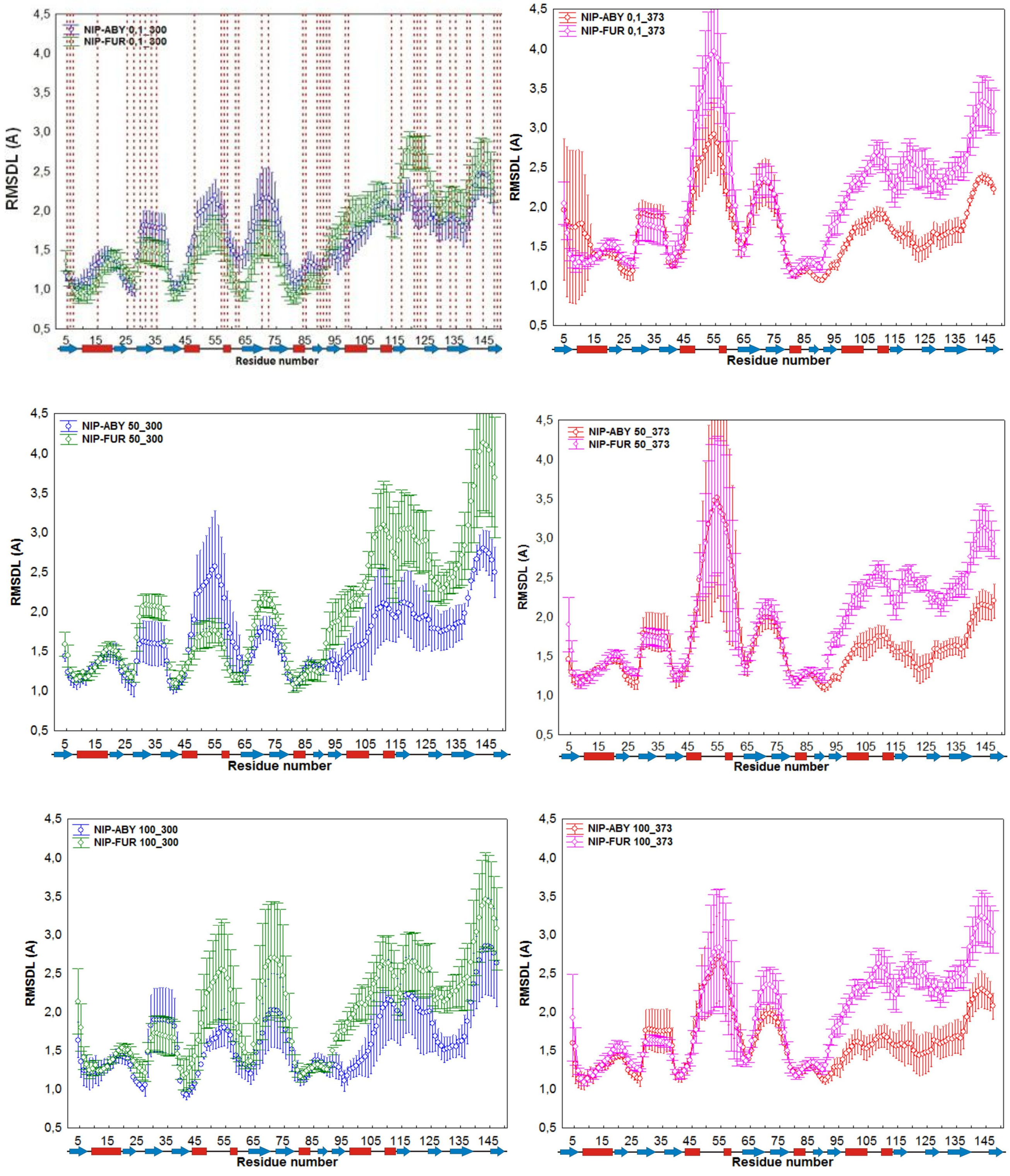

Figure S1: Analysis of the RMSDL for residues in NIP7-ABY and NIP7-FUR models at different temperatures and pressures. (A) Graphs showing the average RMSDL values for residues of the NIP7-ABY (blue) and NIP7-FUR (green) models at 300K. Red dotted lines show positions with amino acid substitutions in *P.furiosus* relative to *P.abyssi* protein. **(B)** Graphs showing the average RMSDL values for residues of the NIP7-ABY (red) and NIP7-FUR (magenta) models at 373K.

Whiskers indicate the 95% confidence intervals. The protein secondary structures are given at the bottom of the graphs: the β-sheets, blue arrows; the α-helices, red rectangles.

A

```
          1        10        20        30        40        50        60        70        80        90        100       110       120       130       140       150 155
          ....|....|....|....|....|....|....|....|....|....|....|....|....|....|....|....|....|....|....|....|....|....|....|....|....|....|....|....|....|....|....|
P.Abyssi  LRVRRASSWELDLILKEAEKYGELLHEFFCVVEGKYRDVYAVNEEVWKIIEDINMRPYSLGTFVGTIRVDENLVEKFYPNLEFFSLIKLEKNYVILGPKASFLFTTGKDAPKEAVREIKWQGSKRVVVLNDLGDIIGIGLINPKSDRRFIKNLKD
P.Abyssi  *EEEE**HHHHHHHHHHHHTTEEE****EEEEESSSEEEEE**HHHHHHTTTS***GGGT*EEEEEEEE*TTS*EEEEE*HHHHTTEEE*SSEEEE*HHHHHHHHTT**B*GGGEEEEE*SS*SEEEEE*TTS*EEEEEEE*TT*STTSEEE***
0,1_300   *EEEE**HHHHHHHHHHHTTTEEE****EEEEESSSEEEEE**HHHHHHTTTS***GGGS*EEEEEEEE*TTS*EEEEE*GGGGGGEEE*SSEEEE*HHHHHHHHTT**B*GGGEEEEE*SS*SEEEEEETTS*EEEEEEE*TT*STS*EEE***
0,1_373   *EEEE**HHHHHHHHHHHTTTEEE****EEEEESSSEEEEE**HHHHTTTTTS***GGGS*EEEEEEEE*TTS*EEEEE*GGGGGGEEE*SSEEEE*HHHHHHHHTT**B*GGGEEEEE*SS*SEEEEEETTS*EEEEEEE*TT*STS*EEE***
50_300    *EEEE**HHHHHHHHHHHTTTEEE****EEEEESSSEEEEE**HHHHTTTTTS***GGGS*EEEEEEEE*TTSSEEEEE*GGGGGGEEE*SSEEEE*HHHHHHHHTT**B*GGGEEEEE*SS*S*EEEEETTS*EEEEEEE*TT*STT*EEE***
50_373    *EEEE**HHHHHHHHHHHTTTEEE****EEEEESSSEEEEE**HHHHTTTTTS***GGGS*EEEEEEEE*TTSSEEEEE*GGGGGGEEE*SSEEEE*HHHHHHHHTT**B*GGGEEEEE*SS*S*EEEEETTS*EEEEEEE*TT*STT*EEE***
100_300   **EEE**HHHHHHHHHHHTTTEEE****EEEEESSSEEEEE**HHHHHHTTT****GGGS*EEEEEEEE*TTSSEEEEE*GGGGGGEEE*SSEEEE*HHHHHHHHTT**B*GGGEEEEE*SS*S*EEEE*TTS*EEEEEEE*TT*STT*EEE***
100_373   **EEE**HHHHHHHHHHHTTTEEE****EEEEESSSEEEEE**HHHHTTTTTS***GGGS*EEEEEEEE*TTS*EEEEE*GGGGGGEEE*SSEEEE*HHHHHHHHTT**B*GGGEEEEE*SS*S*EEEEETTS*EEEEEEE*TT*STT*EEE***
```

B

```
          1        10        20        30        40        50        60        70        80        90        100       110       120       130       140       150 155
          ....|....|....|....|....|....|....|....|....|....|....|....|....|....|....|....|....|....|....|....|....|....|....|....|....|....|....|....|....|....|....|
P.FuriosusIIARRASSWEIDLILKEAEKFGTLVHNFFAIVEGKYRDVYAVNNEVWKIIEDLTVRPFALGTFVGMIKVDENLVEKFYPAIEFFTFVDIEKNYAVLGPKASFLFTTGKDVPKKAVRKLVWKGSKKIVVMNELGDVLGIGRINPSNEEKFIKNITD
P.Furiosus***EB**HHHHHHHHHHHHTTEEE****EEEEESSSEEEEE**HHHHHHHHHS***GGGT*EEEEEEEE*TTS*EEEEE*HHHHTTEEE*SSEEEE*HHHHHHHHTT**B*GGGEEEEE*SS*SEEEEE*TTS*EEEEEEE*TT*STTSEEE***
0,1_300   *EEEE**HHHHHHHHHHHTTTEEE****EEEEESSSEEEEE**HHHHHHTTTS***GGGS*EEEEEEEE*TTSSEEEEE*GGGGGSEEE*SSEEEE*HHHHHHHHTT**B*GGGEEEEE*SS*S*EEEEETTS*EEEEEEE*SS*STS*EEE***
0,1_373   **EEE**HHHHHHHHHHHTTTEEE****EEEEESSSEEEEE**HHHHTTTTTS****GGT*EEEEEEEE*TTS*EEEEE*GGGGGSEEE*SSEEEE*HHHHHHHHTT**B*GGGEEEEE*SS*S*EEEEETTS*EEEEEEE*TT*SSS*EEE***
50_300    *EEEE**HHHHHHHHHHHTTTEEE****EEEEESSSEEEEE**HHHHHHTTTS***GGGS*EEEEEEEE*TTSSEEEEE*GGGGGSEEE*SSEEEE*HHHHHHHHTT**B*GGGEEEEE*SS*S*EEEEETTS*EEEEEEE*SS*STS*EEE***
50_373    *EEEE**HHHHHHHHHHHTTTEEE****EEEEESSSEEEEE**HHHHTTTTTS***GGG*SEEEEEEEE*TTSSEEEEE*GGGGGSEEE*SSEEEE*HHHHHHHHTT**B*GGGEEEEE*SS*S*EEEEETTS*EEEEEEE*TT*SSS*EEE***
100_300   **EEE**HHHHHHHHHHHTTTEEE****EEEEESSSEEEEE**HHHHHHTTTS***GGGS*EEEEEEEE*TTSSEEEEE*GGGGGSEEE*SSEEEE*HHHHHHHHTT**B*GGGEEEEE*SS*S*EEEEETTS*EEEEEEE*SS*SSS*EEE***
100_373   *EEEE**HHHHHHHHHHHTTTEEE****EEEEESSSEEEEE**HHHHTTTTTS***GGG**EEEEEEEE*TTS*EEEEE*GGGGGSEEE*SSEEEE*HHHHHHHHTT**B*GGGEEEEE*SS*S*EEEEETTS*EEEEEEE*TT*SSS*EEE***
```

Figure S2: Analysis of the secondary structure for NIP7-ABY and NIP7-FUR models. (A) NIP7-ABY secondary structure during the MD simulations at different temperatures and pressures. **(B)** NIP7-FUR secondary structure during the MD simulations at different temperatures and pressures.
The most frequent state of the secondary structure is represented for each residue. Regions with the most variable secondary structure in all trajectory runs according to PVS server (http://imed.med.ucm.es/PVS/) shown in grey. Designation of the secondary structure elements: H - alpha helix, B - residue in isolated beta-bridge, E - extended strand, participates in beta ladder, G - 3-helix (3/10 helix), T - hydrogen bonded turn, S – bend, * - coil.

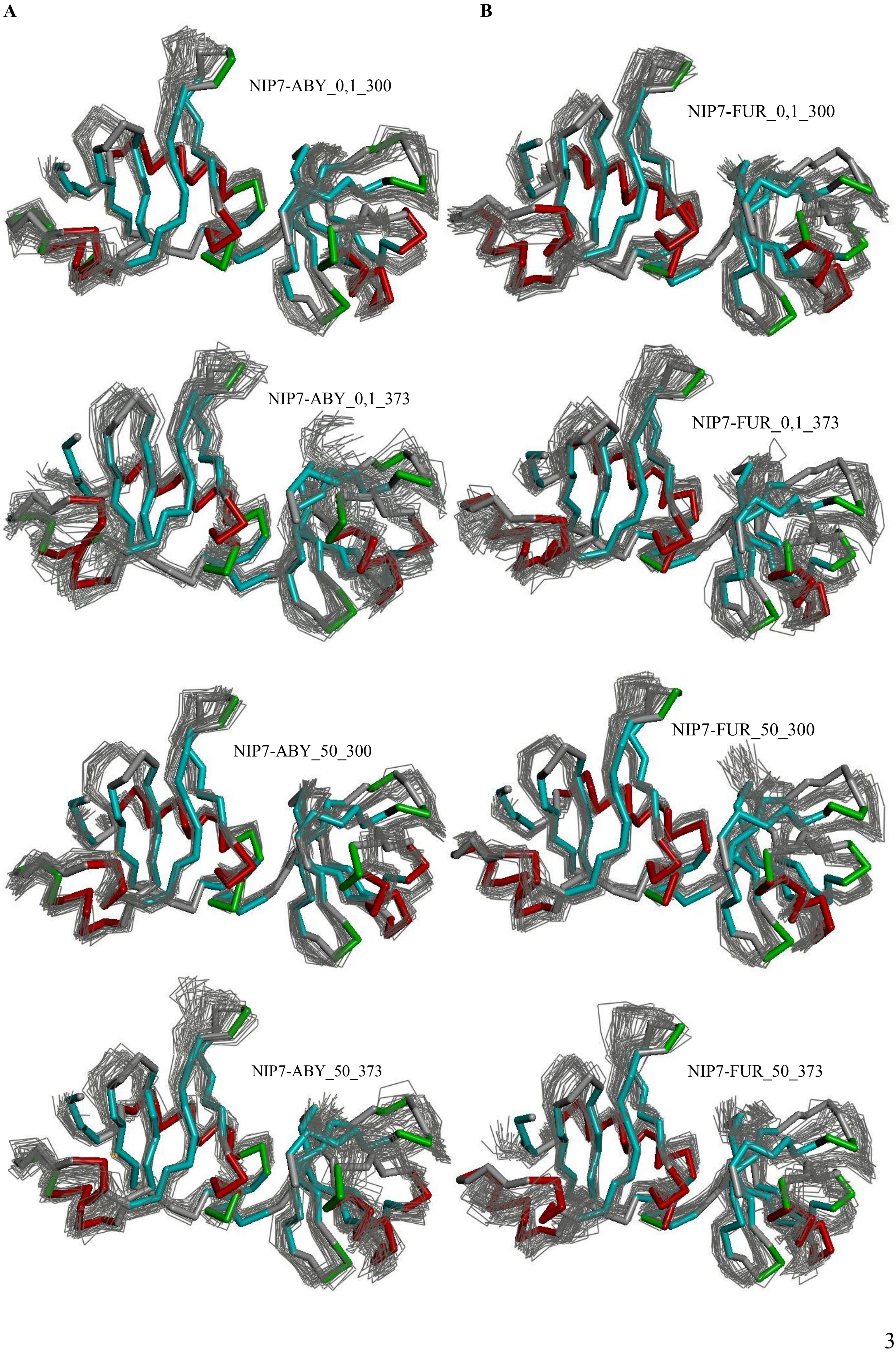
A
B
NIP7-ABY_0,1_300
NIP7-FUR_0,1_300
NIP7-ABY_0,1_373
NIP7-FUR_0,1_373
NIP7-ABY_50_300
NIP7-FUR_50_300
NIP7-ABY_50_373
NIP7-FUR_50_373

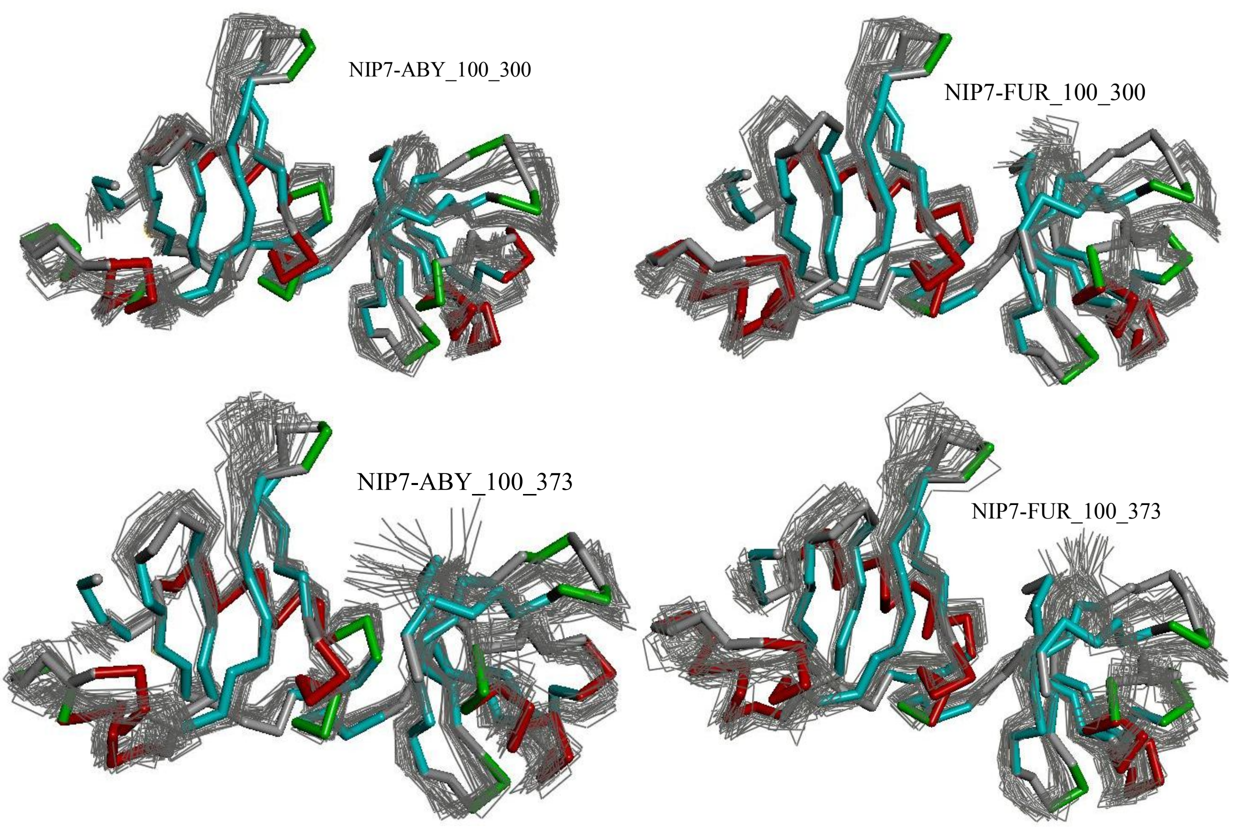

Figure S3: Snapshots of the Cα traces of the NIP7-ABY and NIP7-FUR models at different temperatures and pressures. (A) Snapshots of the NIP7-ABY model. (B) Snapshots of the NIP7-FUR model.
The *P.abyssi* Nip7 crystal structure (PDB ID 2P38:A) is shown in color tube; beta strands, blue; alpha-helices, red; turns, green. Models obtained by the MD simulations are indicated by grey lines.

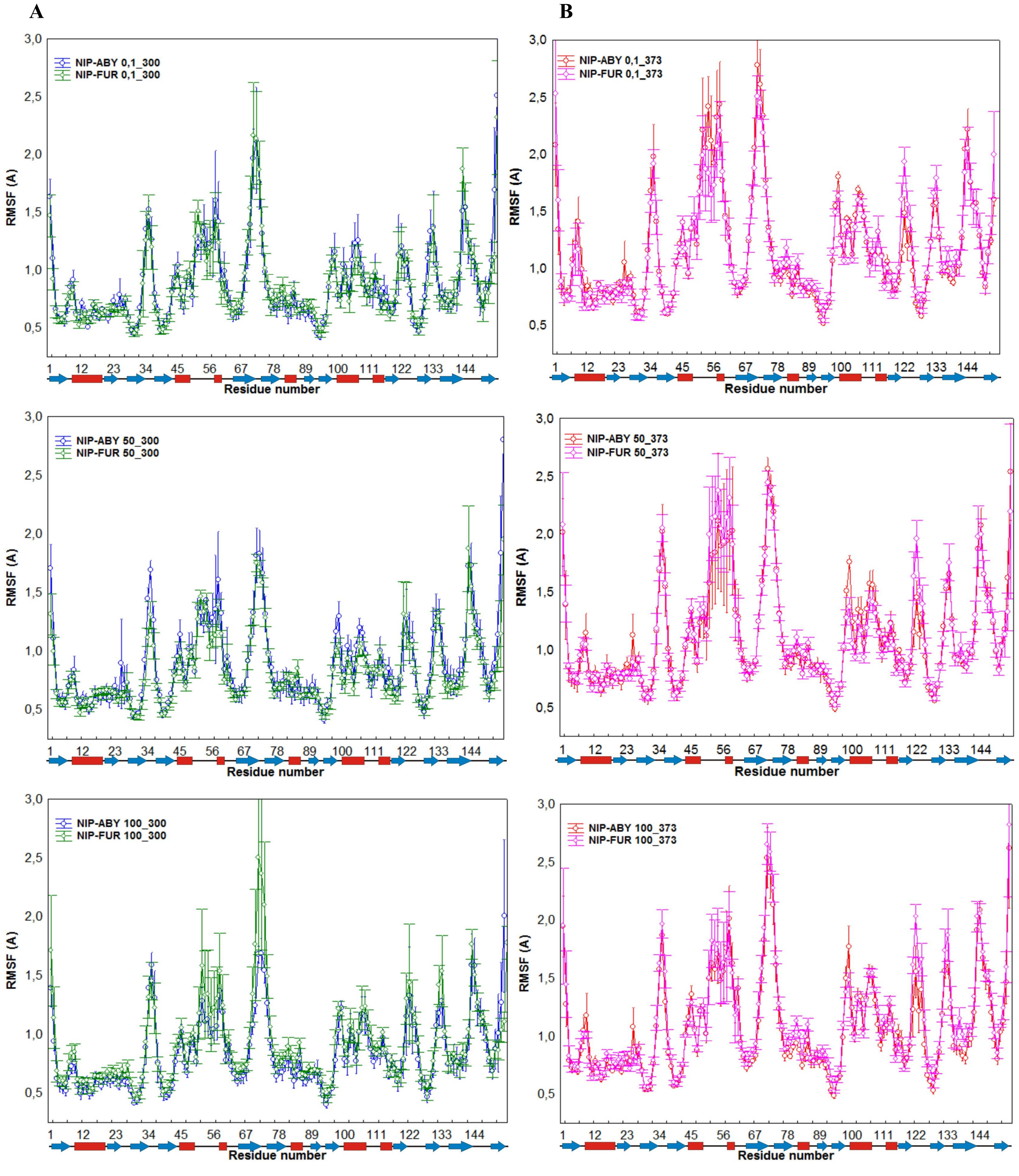

Figure S4: Analysis of the Cα RMSF for NIP7-ABY and NIP7-FUR models. (A) Graphs showing the average RMSF values for residues of the NIP7-ABY (blue) and NIP7-FUR (green) models at 300K. **(B)** Graphs showing the average RMSF values for residues of the NIP7-ABY (red) and NIP7-FUR (magenta) models at 373K.

Whiskers indicate the 95% confidence intervals. The protein secondary structures are given at the bottom of the graphs: the β-sheets, blue arrows; the α-helices, red rectangles.
